# Hypokalaemia and Renal Tubular Acidosis due to Abuse of Nurofen Plus

**DOI:** 10.1155/2012/141505

**Published:** 2012-08-29

**Authors:** M. J. Blackstock, A. Lee

**Affiliations:** Critical Care, Anaesthesia and Pain Management Department, Royal Infirmary of Edinburgh 51 Little France Crescent, Edinburgh EH16 4SA, UK

## Abstract

Nurofen Plus is a common analgesic containing ibuprofen and codeine. We present a case of a 38-year-old lady who developed renal tubular acidosis with severe hypokalaemia, after chronic abuse of Nurofen Plus tablets. She presented with confusion and profound biochemical abnormalities requiring critical care admission for electrolyte replacement. Ibuprofen causes renal tubular acidosis due to its effects on carbonic anhydrase activity.

## 1. Introduction

Nurofen Plus is an over-the-counter analgesic which contains 200 mg ibuprofen and 12.8 mg codeine phosphate per tablet. We report the case of a patient who was admitted to our department with life-threatening hypokalaemia after prolonged ingestion of large amounts of Nurofen Plus.

## 2. Case Report

A 38-year-old woman presented to the emergency department with a two-day history of confusion, agitation, and “restless, swollen legs.” Her past medical history included gastric bypass surgery, depression, and previous alcohol abuse. Regular medications were fluoxetine, omeprazole, and cetirizine. On initial assessment in the emergency room she was tachycardic at 102 beats per minute, her blood pressure was 130/70 mmHg, and her chest was clear to auscultation with an SpO_2_ of 100% on room air. She was confused, with a GCS score of 10 (E_3_V_2 _M_5_), but with no focal neurological abnormalities or rash. Her temperature was 37.5°C and blood sugar was 5.6 mmol/L. Due to her confusion and agitation, sedation was given and a CT brain scan performed. This demonstrated no acute abnormality. A lumbar puncture was performed and treatment for CNS infection instituted with ceftriaxone and acyclovir. Her potassium was 1.9 mmol/L (3.6–5.0 mmol/L), sodium 146 mmol/L (136–145 mmol/L), phosphate 0.57 mmol/L (0.8–1.4 mmol/L), and chloride 122 mmol/L (96–106 mmol/L). Her renal function was otherwise normal with a urea of 2.7 mmol/L (2.5–6.6 mmol/L) and creatinine 56 *μ*mol/L (60–120 *μ*mol/L). An arterial blood gas (FiO_2_ 0.40) demonstrated a compensated metabolic acidosis: H^+^ 38.6 nmol/L, PaCO_2_ 3.5 kPa, PaO_2_ 24.44 kPa, HCO_3_ 18.8 mmol/L, BE 8.2 mmol/L, Lactate 1.13 mmol/L. An ECG showed ST segment depression and U-waves but no ectopic beats or arrhythmias.

On further questioning, the patient's family suggested that she may have been taking large amounts of Nurofen Plus, as this had been an issue previously.

Due to her confusion and profound biochemical abnormalities she was admitted to the intensive care unit for further management. 

Her management on ITU involved invasive monitoring and correction of the biochemical abnormalities. During the initial 48 hours in hospital she received 509.5 mmol of intravenous potassium replacement, which increased the serum potassium to 4.8 mmol/L. Phosphate replacement was given and once the serum potassium level had improved, 1.26% sodium bicarbonate was used as maintenance intravenous fluid. With the correction of her biochemical abnormalities (Table 1), her neurological function improved to GCS 15 (E_4_V_5 _M_6_). The biochemical abnormalities in the serum and urine suggested a diagnosis of renal tubular acidosis with a serum anion gap of 9 mmol/L, urinary anion gap of 20 mmol/L, and transtubular potassium gradient of 11.37. On improvement of her symptoms and conscious level, the patient admitted to taking large amounts of Nurofen Plus tablets (20–40 per day) during the preceding weeks.

## 3. Discussion

In a small number of cases, deliberate overdose of Nurofen Plus has been reported as causing hypokalaemia from type two proximal renal tubular acidosis [1–5]. The biochemical abnormalities of hypokalaemia, hyperchloraemia, hypophosphataemia, and metabolic acidosis are consistent with those found in our patient. Ibuprofen (2-(4-isobutylphenyl)propionic acid) is a commonly used and readily available analgesic. In overdose, toxicity resulting in serious morbidity or mortality is rare, although deaths from ibuprofen overdose do occur [6, 7]. 

In our patient, the prolonged and excessive intake of Nurofen Plus was likely due to the codeine component of the preparation, resulting in opiate dependence. 

Type two renal tubular acidosis has multiple causes and results in a hyperchloraemic metabolic acidosis, hypokalaemia, and hypophosphataemia. Renal tubular acidosis caused by Ibuprofen is not well understood, but is thought to involve inhibition of carbonic anhydrase activity [3]. Carbonic anhydrase (CA) exists as 15 isoforms and CA type two (CAII) is the predominant form (95%) in the kidney [8]. Deficiency of CAII has been demonstrated to cause renal tubular acidosis and nonsteroidal anti-inflammatory drugs (NSAIDs) have been shown to inhibit CAII actions, although the effects of Ibuprofen were less than flurbiprofen [9, 10]. This mechanism may explain why Ibuprofen toxicity has variously been described as causing type two or type four renal tubular acidosis as CAII is distributed throughout most of the nephron and therefore its inhibition may not cause solely a proximal tubular effect. 

Renal tubular acidosis may cause significant hypokalaemia and this occurred in our patient with a life-threatening hypokalaemia of 1.8 mmol/L. Serum measurement of potassium is a relatively poor reflection of total body potassium which is approximately 3.5 moles in the average adult, 98% of which is intracellular. The total body potassium is altered by age, sex, weight, and ethnicity [11, 12]. Our patient had a total body potassium deficit of approximately 20%, estimated from her requirement for potassium replacement. 

Management of renal tubular acidosis secondary to Ibuprofen involves removal of the precipitating drug, correction of the biochemical abnormalities, and supportive care. 

Combination analgesics containing an opioid component are commonly used and are readily available in many countries for the management of acute or chronic pain [13]. Addiction to the opioid component leading to an increase in self-administration has led to life-threatening and fatal consequences from the non-opioid component. Inadvertent self-poisoning in this manner has been widely reported as a cause of liver failure from acetaminophen, leading to a requirement for liver transplantation or death [14]. This has recently prompted the US Food and Drug Administration (FDA) to limit the amount of acetaminophen permitted in a combination tablet to 375 mg [15].

Medical practitioners need to have a greater awareness of the hazards associated with opioid-containing analgesics, in particular accidental poisoning with the non-opioid component. This case represents a rare but important effect of Ibuprofen toxicity and combination analgesics.

## Figures and Tables

**Table 1:** Biochemical results.

Hours	Urea (mmol/L)	Creat (*μ*mol/L)	Na (mmol/L)	K (mmol/L)	HCO_3_ (mmol/L)	Cl (mmol/L)	PO_4_ (mmol/L)	Mg^2+^ (mmol/L)	BE (mmol/L)	H^+^ (nmol/L)
0	2.7	56	146	1.9	13		0.57	0.93	−7.4	40.7
5	2.9	48	149	1.8	16	124	0.65	0.78	−8.2	38.6
9	2.7	52	148	2.3	15	125	0.71	0.82	−8.1	33.9
17	2.5	50	149	2.3	15	123	1.10	0.78	−7.3	34.6
41	1.5	46	142	4.8	15	123	0.93	0.71	−9.1	41.3
